# Phase 1 Trial of *Withania somnifera* Leaf Extract (RH324) in Advanced Non-Small Cell Lung Cancer Including [^18^F]FDG PET/CT as a Short-Term Metabolic Biomarker to Assess Efficacy: A Novel Model for Assessment of Complimentary Therapies in Early Phase Human Clinical Trials

**DOI:** 10.1177/15347354251410182

**Published:** 2026-02-13

**Authors:** Jin Uk Heo, Santosh Rao, Herbert B. Newton, Afshin Dowlati, Raymond F. Muzic, Arash Kardan

**Affiliations:** 1 Department of Radiology, Case Western Reserve University and University Hospitals Cleveland Medical Center, OH, USA; 2 Department of Biomedical Engineering, Case Western Reserve University and University Hospitals Cleveland Medical Center, OH, USA; 3 Seidman Cancer Center, University Hospitals, Case Western Reserve University, Cleveland, OH, USA; 4 Division of Nuclear Medicine and Molecular Imaging, Department of Radiology, Baylor College of Medicine, Houston, TX, USA

**Keywords:** integrative oncology, phase 1 human trial, positron emission tomography, FDG, lung cancer, *Withania somnifera*

## Abstract

**Background:**

*Withania somnifera* (WS), commonly known as ashwagandha, has been used in the traditional medical system of India. It has shown significant activity against numerous solid tumor varieties in pre-clinical in vitro and in vivo studies. This study focuses on RH324 (ReHeva Biosciences, Columbus, OH), a pharmaceutical-grade formulation derived from WS, which has received FDA allowance for clinical development as a botanical drug in the treatment of cancer.

**Methods:**

A phase 1 open label dose ranging study of oral RH324 in advanced non-small cell lung cancer (NSCLC) was conducted. The primary endpoint of the study was assessment of short-term safety and tolerability. An exploratory aim was assessment of RH324’s anti-neoplastic effects as measured by changes in tumor metabolism using [^18^F]-labeled 2-fluoro-2-deoxy-D-glucose (FDG)-PET/CT scans performed before and after completing a 28-day RH324 monotherapy regimen. A total of 9 patients were enrolled, 5 of which completed the trial, including imaging, and were evaluable.

**Results:**

All study safety and tolerability primary endpoints were met. Analysis revealed notable response rates across different SUV parameters (SUV_max_, SUV_mean_, and SUV_peak_) and there were no new metastatic lesions identified, further supporting RH324’s anti-neoplastic effects.

**Conclusion:**

RH324 was safe and well tolerated as a monotherapy in advanced NSCLC. FDG-PET/CT provided a novel methodology as a short-term metabolic endpoint with an in vivo biomarker assessment of potential clinical efficacy using a metabolic surrogate of disease activity. This approach offered deeper insights into tumor metabolism and heterogeneity, underscoring the potential of RH324’s anti-neoplastic effects in treating refractory NSCLC.

**Clinical Trial Registration:**

ClinicalTrials.gov, Identifier: NCT05580172, Registered on October 17, 2022.

## Introduction

*Withania somnifera* (WS), commonly known as ashwagandha, has been used in the traditional medical system of India—Ayurveda—for thousands of years, and is one of the most important herbal therapies.^1,2^ Over the past 20 years, ethnopharmacological and phytochemical analyses have begun to clarify the bioactive compounds in WS that can mediate potential clinical benefits, including potent anti-cancer activity. The most bioactive compounds are the with anolides, large multi-cyclic, 28-carbon chemicals with active oxygen groups to interact with proteins and other large molecules in tumor cells.^2,3^ Withaferin A (WFA) is the most extensively studied bioactive compound found in WS, and it has shown significant activity against numerous solid tumor varieties in pre-clinical in vitro and in vivo studies, including glioblastoma.^2^ Other withanolides with significant anti-cancer activity include withanone, withanolide A, and 4β-hydroxywithanolide E.

Despite the well-documented traditional use of WS, its anti-cancer mechanisms remain only partially elucidated, involving interactions across a broad spectrum of molecular targets. The scientific literature, exceeding 1700 articles on WS, details its immunomodulatory, anti-inflammatory, and neuroprotective properties, among others. However, the exploration of WS in cancer therapy is limited, as evidenced by a relatively modest collection of 70 articles which suggest significant potential through restorative, radiosensitizing, and anticancer effects in preclinical models. It is well established that WS roots have a long history of use in Ayurvedic medicine and are generally regarded as safe when consumed at recommended doses. By contrast, reports on WS leaves have been more variable. The difference in phytochemical composition—roots being relatively lower in steroidal alkaloids compared to leaves—underpins much of the perceived disparity in safety between the 2 plant parts. In our case, RH324 (the WS extract evaluated in this study) was developed as a proprietary full-spectrum extract, formulated to retain a natural mixture of alkaloids while ensuring a consistent and reproducible composition.

Experience with WS in the clinic is limited with several studies focusing on quality of life. Notably, a phase II trial assessed the impact of WS on chemotherapy-induced fatigue in breast cancer patients, revealing statistically significant improvements in fatigue and quality of life.^4^ Another study, a phases I to II trial in osteosarcoma patients, is exploring WS’s pharmacokinetics and therapeutic effects, including quality of life and toxicity (ClinicalTrials.gov Identifier: NCT00689195).^5,6^ Additionally, a double-blind, placebo-controlled phase II trial aims to evaluate WS’s potential to alleviate “chemo fog” in cancer patients undergoing chemotherapy (ClinicalTrials.gov Identifier: NCT04092647).^7^

The current study focuses on RH324 (ReHeva Biosciences, Columbus, OH), a pharmaceutical-grade formulation derived from WS, which has received FDA allowance for clinical development as a botanical drug for the treatment of cancer. Preclinical in vitro and in vivo research indicates that A549 non-small cell lung cancer (NSCLC) cells exposed to WFA exhibit decreased growth and were sensitized to ionizing radiation.^8-11^ Moreover, xenograft studies of A549 human lung adenocarcinoma cells have demonstrated dose-dependent cytotoxicity in response to WFA in A549 cells, while non-carcinoma control cell lines WI-38 and PBMC were unaffected. Moreover, the authors further elucidated the underlying anti-cancer mechanisms by demonstrating involvement of reactive oxygen species in WFA-mediated cytotoxicity against cancer cells.^10,12^

These demonstrated in vitro antineoplastic activities motivate the current in vivo exploratory positron emission tomography and computed tomography (PET/CT) analysis of RH324 as a standalone therapy for non-small cell lung cancer.

NSCLC remains a difficult-to-treat malignancy. Patients with metastatic disease ultimately exhibit disease progression after standard treatment with either a molecular targeted therapy, chemotherapy or immunotherapy. Compounds utilized in traditional medical systems have not been well studied in this setting. Furthermore, large clinical trials with long-term statistical endpoints such as progression-free and overall survival are both time and resource consuming limitations. Furthermore, large clinical trials with long-term statistical endpoints such as progression-free and overall survival are both time- and resource-consuming. As the therapeutic window for traditional therapies is quite wide based on thousands of years of experience, classic drug development strategies of dose exploration may not be of value. In this study we report the first NSCLC anti-tumor assessment using a short-term metabolic endpoint for RH324.

## Method

### Overview

This is a phase 1 open label dose ranging study of oral RH324 in advanced non-small cell lung cancer. This study was approved by the University Hospitals Clevland Medical Center, Submission number MOD00025164 in November 2022. All participants provided written informed consent before enrollment. The primary endpoint of the study was assessing the short-term (28 days) safety and tolerability of oral RH324 in patients with advanced NSCLC as measured by the incidence of adverse events as defined by the NCI Common Terminology Criteria for Adverse Events (CTCAE). A key exploratory aim was to assess RH324’s anti-neoplastic effects as measured by changes in tumor metabolism by employing [^18^F]-labeled 2-fluoro-2-deoxy-D-glucose (FDG)-PET/CT scans before and after the completion of the 28-day RH324 monotherapy regimen.

### Investigational Product

RH324 is an investigational botanical drug being developed under a U.S. FDA Investigational New Drug (IND) application. The clinical study drug product is the RH324 botanical drug product comprising RH324 botanical drug substance filled into hard, opaque vegetarian capsules (Vcaps, Capsugel, USA), size 00, color Swedish Orange; each capsule contains 500 mg of RH324. Detailed quantitative composition and chromatographic profiles are proprietary trade secrets and are not publicly disclosed in this manuscript. All drug substance information including strength, composition, and detailed characterization has been fully disclosed to the FDA within the IND Chemistry, Manufacturing, and Controls package. The investigational product has been rigorously characterized and evaluated.

### Data Acquisition

The study was conducted under an IRB-approved phase 1 clinical trial (ClinicalTrials.gov Identifier: NCT05580172) at University Hospitals Seidman Cancer Center and Case Western Reserve University.^13^ Inclusion criteria included adults over 18 years with measurable disease according to Response Evaluation Criteria in Solid Tumors (RECIST) 1.1 with advanced NSCLC who have failed all standard of care treatments including chemotherapy, targeted therapy, and immunotherapy.^14^ Patients must have a performance status (Eastern Cooperative Oncology Group) ≤2 at the time of enrollment with a life expectancy >2 months. Minimum baseline laboratory values for study inclusion included: hemoglobin ≥9 g/dL, neutrophils >1000/µL, platelets >50 000/µL, liver function tests less than or equal to twice upper limit of normal, serum creatinine ≤2 mg/dL, creatinine clearance ≥30 mL/minute, hemoglobin A1C <7, and normal thyroid function.

Exclusion criteria included prior use of *Withania somnifera*, prior diagnosis of phenylketonuria, inability to swallow capsules, hypersensitivity to study drug ingredients, unstable medical or surgical condition, history of additional cardiac risk factors, requiring drugs that are “strong” inhibitors of cytochrome P450, requiring irradiation, requiring intravenous fluids or hyperalimentation, requiring transfusions or dialysis, active infection (including human immunodeficiency virus), must exceed washout period of prior treatments at the time of regimen initiation, and preexisting psychiatric, neurological, or other condition that precludes subject’s ability to participate in the trial.

Participants in each of the 3 dosing groups received the specified dose of RH324 administered orally twice daily for a 28-day cycle. Five participants underwent FDG-PET/CT imaging at baseline and upon completion of the 28-day RH324 monotherapy regimen. Each PET/CT scan was acquired ~60 minutes after injection for torso field-of-view (base of the skull to mid-thigh) with arms positioned alongside the body. A 30-second, low-dose, 120-kVp non-contrast CT scan was performed. FDG images were obtained using the scanner manufacturer’s BLOB-based ordered subsets, time-of-flight reconstruction with relaxation parameter set to 1.0.

### Image Data Analysis

MIM Maestro version 7.0.66.6.10 (MIM Software, Cleveland, OH) was used to contour the targets and acquire all SUV measurements described in Section 2.3.1. The workflow for delineating the target contours was as follows: (1) Navigate to the center (approximately) axial slice of the target, as determined by clinical experts. (2) Select the PET Edge tool and click on the approximate center voxel. (3) Drag the PET Edge tool along the seemingly longest axis. (4) Release the click, which generates the initial contour. (5) Perform MIM Maestro’s “Smooth” operation twice. (6) The resulting contour is the final target contour.

This semi-automated contouring method was chosen to minimize human error and reduce inter- and intra-operator variability, as opposed to fully manual contouring. All contours were confirmed by the attending nuclear medicine physician. MIM Maestro’s provided default SUV_mean_, SUV_max_, and SUV_peak_ values were then used to extract the SUV measurements. These contours and images were subsequently used to determine evaluable targets, which is described in detail in Section 2.3.2, and exported for partial volume correction, which is described in detail in Section 2.3.3.

#### Metabolic Response Assessment

The metabolic response to RH324 treatment was quantitatively assessed by calculating the percentage change in standardized uptake value (SUV) from pre-treatment and post-treatment FDG-PET/CT scans:

$$Relative\;\;Difference\;(\%)=\frac{SUV_{post}-SUV_{pre}}{SUV_{pre}}\times 100\%$$

Here, the SUV normalization is by body mass, so the values have implicit units of g/ml.

In these patients, untreated lesions would be expected to show a prominent SUV increase, especially those with high initial glucose metabolic activity. In our study, a lesion without a prominent post-treatment SUV increase was interpreted as a positive response to the monotherapy. We defined progression as a ≥30% increase in SUV, while a ≤15% increase, including a decrease in SUV (indicative of a negative percentage change), was indicative of a response to our monotherapy. Changes in SUV between 15% and 30% were considered indeterminate, as they did not provide a clear indication of response or progression. These thresholds were in alignment with PERCIST guidelines, ensuring the consistency of our methodology and its adherence to recognized standards for evaluating tumor response via PET imaging.^15^

Specifically, this study used SUV_mean_, SUV_max_, and SUV_peak_. SUV_mean_ as a measure of metabolic activity across the tumor, providing an overview of its metabolic load. SUV_max_, indicating the maximum SUV within the tumor, identifies the most metabolically active regions, crucial for assessing aggressive tumor behavior and treatment response. SUV_peak_, calculated as the mean within an ~1.2 cm diameter (1 mL volume) sphere centered at the tumor’s most active part, offers a balanced metric that combines high metabolic activity focus with reduced noise and heterogeneity impact, ensuring a reliable assessment of peak metabolic activity. These metrics collectively enable a comprehensive evaluation of the treatment’s effect on tumor metabolism.

#### Target Definitions

In the comprehensive evaluation of all potential regions of interest (ROI) identified within the cohort, a total of 23 targets were initially identified. However, 7 were excluded due to their dimensions being smaller than 3 × 3 × 3 voxels in FDG image, corresponding to a volume of 1.728 mL, leaving 16 targets classified as evaluable targets for assessment in this study. These encompassed both primary NSCLC lesions and metastatic sites as verified by clinical experts.

Within this subset of evaluable targets, lesions exhibiting a SUV_max_ of 7.5 g/mL or higher were categorized as FDG-avid targets. The designation of FDG-avid targets was predicated on the rationale that these lesions exhibited the highest metabolic activity, indicative of significant viability, aggressive pathology, and an elevated risk of rapid progression, in stark contrast to lesions manifesting lower initial SUV_max_ values.

#### Partial Volume Correction

The partial volume effect (PVE) poses a challenge in PET imaging due to the limited spatial resolution of PET systems, leading to signal blending from adjacent structures. This is particularly evident in small lesions, where the actual signal spreads beyond the object’s size, causing an underestimation of peak activity concentrations. Correcting for PVE, known as partial volume correction (PVC), is vital for accurate PET quantification, ensuring that measurements reflect the true metabolic activity of the tissues.

In our study, the Richardson-Lucy (RL) algorithm was chosen to produce a PVC PET image using MATLAB (ver 23.2.0.2428915).^16,17^ It is an iterative procedure for recovering an image that has been blurred by a known point spread function (PSF), analogous to the PVE in PET imaging. The RL algorithm can be applied to correct the PVE, enhancing image clarity and improving quantitative accuracy. In our approach to PVC using the RL deconvolution algorithm, we calibrated the procedure with carefully chosen parameters to effectively mitigate the PVE inherent in PET imaging. The iterative deconvolution was set to 5 iterations, balancing correction precision with computational demand. A damping parameter of 0.1 was employed to control iteration stability and reduce noise amplification. The subsampling factor was maintained at 1, ensuring the preservation of the original image resolution. For the deconvolution kernel, we utilized a 3D Gaussian PSF with a full width at half maximum (FWHM) of 4 mm and a kernel size of 11 pixels, parameters chosen to align with the spatial resolution characteristics of our PET system. This PVC process was integral to refining PET quantification, aiming to produce a more accurate representation of metabolic activity within the targeted lesions.

## Results

A total of 35 patients were screened. Of these 8 were “screen failures” due to use of “nonprotocol” products. These included 2 subjects who had additional risk factors and prior treatment (insufficient washout period). Eighteen subjects chose to participate in another study. A total of 9 patients were enrolled, 5 of which completed the imaging part of the trial and were evaluable. All 9 enrolled subjects were female, 5 were White, 3 were Black, and 1 was of mixed race, with a mean age of 62.8 years (29-87 years). All 9 had histopathologically confirmed advanced NSCLC. Of these, 6 patients presented at baseline with Stage 4 disease. Patient characteristics are summarized in Table 1. All the clinical trial participants were female. This outcome was purely by chance given the small sample size of the trial. The inclusion criteria specified enrollment of adults (≥18 years of age), with no gender bias in recruitment.

**Table 1.** Patient Characteristics.

Dose group: (TDD mg)	Number	Age	Sex	Race/ethnicity	ECOG	Current or prior smoker	Study status	FDG-PET imaging
1 (1000)	101	66	F	White	2	Current	Completed	Yes
	102	69	F	White	2	Prior	WD prior to treatment	No
	103	29	F	White	2	Prior/current E cigs	Completed	Yes
2 (2000)	204	45	F	White	2	Prior	Completed	Yes
	205	87	F	White	2	Prior	Completed	Yes
3 (4000)	308	63	F	Black/African American	2	Prior	WD SAE not related	No
	309	64	F	Black/African American	2	Prior	WD SAE not related	No
	310	75	F	Black/African American	1	Prior	Completed	Yes
	311	72	F	White	1	Current	WD, no AE reported	No

All patients had advanced NSCLC—documented by histopathologic confirmation.

Abbreviations: WD, withdrawn; SAE, significant adverse event.

### Safety/Toxicity Primary Endpoints

Of the 9 enrolled in the study, 3 subjects enrolled in dose Level 1 with a total daily dose (TDD) of 1000 mg, of which 1 subject (102) withdrew from the study prior to receiving study drug. The remaining 2 subjects (101, 103) completed the study treatment as planned. For dose Level 2 (TDD 2000 mg), 2 subjects (204, 205) enrolled and both completed the study treatment as planned. For dose Level 3 (TDD 4000 mg), 4 subjects enrolled, however, only 1 (310) completed the study treatment as planned. Two subjects (308, 309) withdrew early due to serious adverse events (SAEs) deemed “not related” to the study drug; the final subject (311) withdrew early without reporting any AEs. This summarized in Table 1.

### Imaging Results

**Figure 1.**
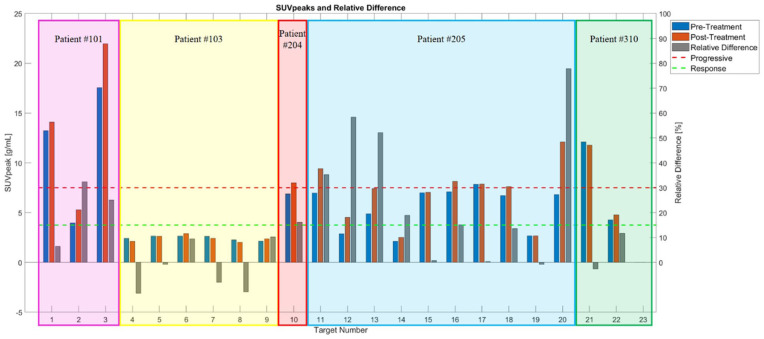
SUV_peak_ and RD for all 23 targets across subjects. SUV_peak_ values from pre- and post-treatment are shown in blue and orange bars, respectively, with the *y*-axis scale on the left. The RD, representing the percentage change in SUV_peak_ between pre- and post-treatment, is displayed in gray bars, with the corresponding *y*-axis on the right. The green dashed line marks the lower threshold, below which a target is considered responsive to our monotherapy. The red dashed line marks the upper threshold, above which a target is classified as progressive. Values of RD falling between these 2 dashed lines indicate changes that are not significant enough to categorize the target as either progressive or responsive, and therefore, categorized as indeterminate. RD, relative difference.

There were no new metastatic lesions detected on post-therapy FDG-PET/CT. In Figure 1, SUV_peak_ and RD data are presented for all 23 targets across subjects. However, targets falling below the evaluable volume threshold of 1.728 mL, specifically targets 2, 10, 11, 13, 20, 21, and 23, were not included in the statistical analysis. For example, in subject #1, target #1 falls below the green dotted line, indicating a response to the monotherapy. Target #2 exceeds the red dotted line, suggesting disease progression; however, it is only 0.1 mL in volume and thus not considered an evaluable target, leading to its exclusion from the statistical analysis. Target #3, whose RD lies between the green and red dotted lines, is categorized as indeterminate. This approach was applied consistently across all 23 targets to determine their evaluability and response categorization. Notably, target 23 was so small that the MIM Maestro software could not calculate its SUV_peak_. The response rates for all 3 metrics (SUV_max_, SUV_mean_, and SUV_peak_) were significantly higher than the combined indeterminate and progressive rates, indicating potential efficacy of RH324 as a standalone therapy for non-small cell lung cancer.

**Figure 2.**
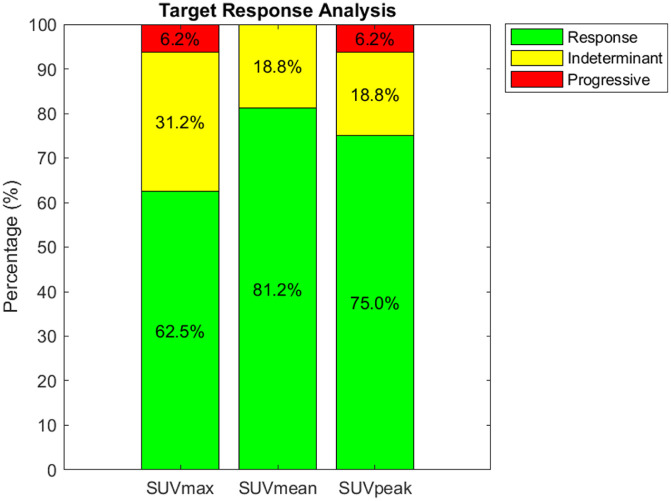
Analysis of SUV percent changes pre- and post-RH324 monotherapy for all evaluable targets that are larger than 1.7 mL. The stacked bar chart illustrates the distribution of treatment response categories based on changes in SUV metrics: SUV_max_, SUV_mean_, and SUV_peak_. The green, yellow, and red segments represent response, indeterminate, and progressive rates, respectively. The data indicate a majority response rate across all metrics, with the highest response observed for SUV_mean_ (81.2%). Indeterminate rates were lowest for SUV_mean_ and SUV_peak_ (18.8%), and progressive disease was identified in 6.2% of cases assessed with both SUV_max_ and SUV_peak_, with no progression observed with SUV_mean_.

Evaluation of SUV percent changes pre- and post-administration of RH324 monotherapy for all evaluable targets, that is, larger than 1.728 mL, is presented in Figure 2. We observe response rates of 62.5%, 81.2%, and 75.0% for SUV_max_, SUV_mean_, and SUV_peak_, respectively. The indeterminate rates are measured at 31.2%, 18.8%, and 18.8%, while progressive rates are noted at 6.2% for both SUV_max_ and SUV_peak_, with no progressive change detected in SUV_mean_.

**Figure 3.**
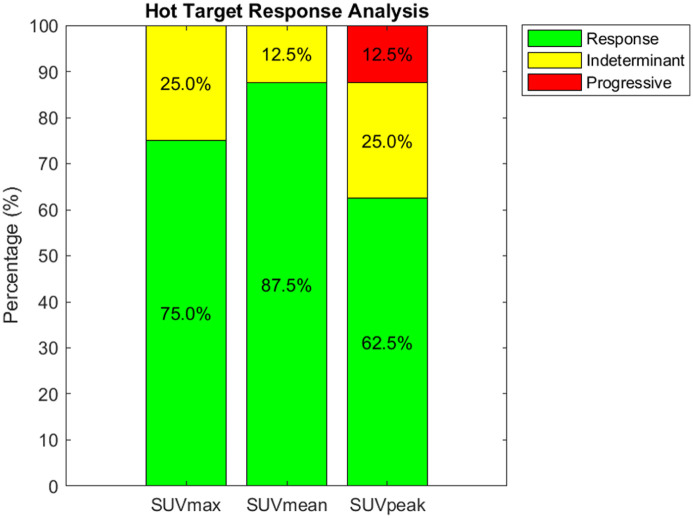
Identical analysis as shown in Figure 2, but for FDG-avid targets exhibiting SUV_max_ higher than 7.5 g/mL. The data indicates a majority response rate across all metrics, with the highest response observed in SUV_mean_ (87.5%). Indeterminate rates were lowest for SUV_mean_ (12.5%), and progressive disease was identified in 12.5% of cases assessed with SUV_peak_, with no progression observed based on SUV_max_ or SUV_mean_.

An identical evaluation of evaluable targets, but restricted to FDG-avid targets that exhibit SUV_max_ higher than 7.5 g/mL is presented in Figure 3. We observe response rates of 75.0%, 87.5%, and 62.5% for SUV_max_, SUV_mean_, and SUV_peak_, respectively. The indeterminate rates are measured at 25.0%, 12.5%, and 25.0%, while a progressive rate is noted at 12.5% for SUV_peak_, with no progressive change detected in SUV_max_ and SUV_mean_. This result indicates that when evaluating only the targets with the highest probability of metastasis, RH324 appears to have a tangible effect in deterring tumor progression, particularly as no progressive changes were observed for SUV_max_ and SUV_mean_ in this subset.

**Figure 4.**
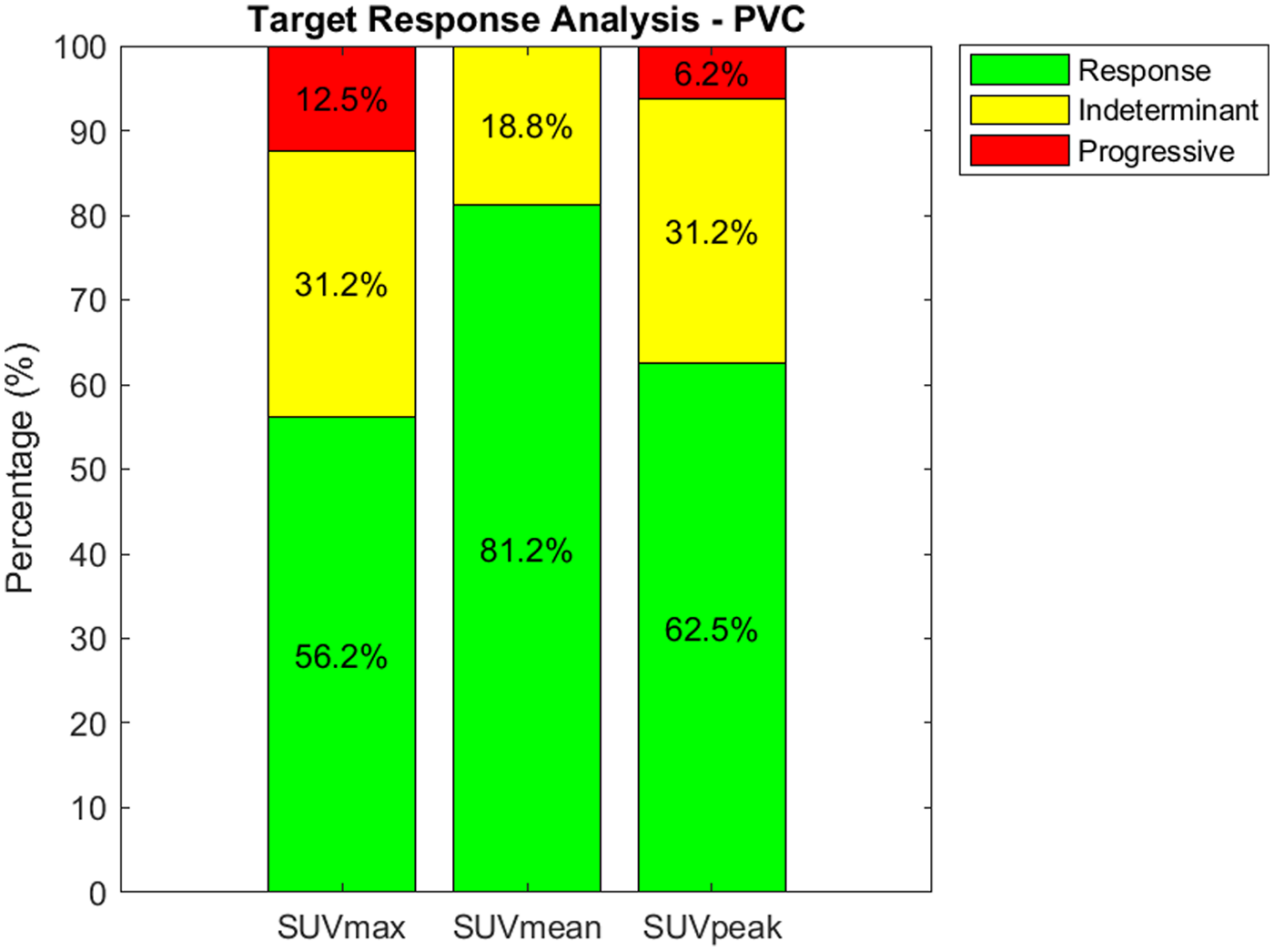
Identical analysis as shown in Figure 2, but after PVC is applied. The data indicates a majority response rate across all metrics, with the highest response observed in SUV_mean_ (81.2%). Indeterminate rates were lowest for SUV_mean_ (18.8%), and progressive disease was identified in 12.5% and 6.2% of cases assessed with SUV_max_ and SUV_peak_, respectively, with no progression observed based on SUV_mean_.

**Figure 5.**
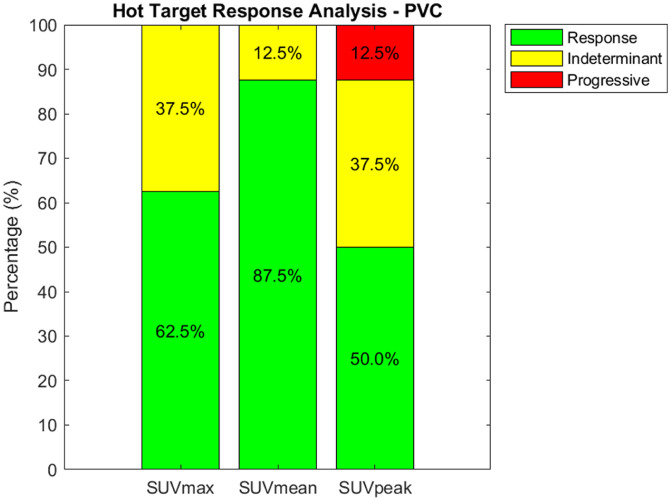
Identical analysis as shown in Figure 3, but after PVC is applied. The data indicates a majority response rate across all metrics, with the highest response observed in SUV_mean_ (87.5%). Indeterminate rates were lowest for SUV_mean_ (12.5%), and progressive disease was identified in 12.5% of cases assessed with SUV_peak_, with no progression observed based on SUV_max_ and SUV_mean_.

The 2 evaluations of evaluable targets without and with SUV selection were also conducted on partial volume corrected images. Results without SUV selection are presented in Figure 4. These achieve a response rate of 56.2%, 81.2%, and 62.5% for SUV_max_, SUV_mean_, and SUV_peak_, respectively. The indeterminate rates are measured at 31.2%, 18.8%, and 31.2%, while progressive rates are noted at 12.5% and 6.2% for SUV_max_ and SUV_peak_, respectively, with no progressive change detected in SUV_mean_. This result suggests that the higher response rates observed in the standard target SUV evaluation are likely not attributable to PVE but rather represent a real effect of RH324. The partial volume corrected SUV analysis still shows higher response rates than indeterminate or progressive outcomes (Figure 5).

Evaluation of evaluable targets with SUV selection after PVC is presented in Figure 4. We observe a response rate of 62.5%, 87.5%, and 50.0% for SUV_max_, SUV_mean_, and SUV_peak_, respectively. The indeterminate rates are measured at 37.5%, 12.5%, and 37.5%, while a progressive rate is noted at 12.5% for SUV_max_, SUV_peak_, with no progressive changes detected in SUV_max_ and SUV_mean_. This result reinforces the earlier observations that RH324 may effectively deter tumor progression even in high-risk metastatic targets, as no progressive changes were seen in SUV_max_ and SUV_mean_ even after PVC.

## Discussion

In this phase 1 escalation study in heavily treated advanced NSCLC patients, treatment with oral RH324 elicited significant metabolic response rates as measured by 28-day post-treatment FDG PET/CT imaging compared with pre-treatment baseline. RH324, a pharmaceutical grade WS therapy (ashwagandha) was well-tolerated with no RH324-related significant adverse events noted. While the study interval was short, it is noteworthy that multiple study participants achieved a response to treatment. It is noteworthy the study population had advanced, metastatic NSCLC refractory to multiple lines of prior therapy. Given the aggressive nature of this refractory disease which demonstrated progression on multiple lines of prior therapy, the responses observed with RH324 would suggest the need for further investigation in this patient population.

Our analysis revealed notable response rates across different SUV parameters (SUV_max_, SUV_mean_, and SUV_peak_) in the evaluation of RH324 monotherapy effectiveness for NSCLC. In particular, we observed high response rates in FDG-avid targets having a high pre-treatment glucose metabolic activity defined as SUV_max_ exceeding 7.5 g/mL. Post-PVC adjustments further strengthened these findings of a measurable metabolic response to RH324 monotherapy, supporting RH324’s anti-neoplastic effects. In addition, there were no new metastatic lesions identified on post monotherapy FDG PET/CT imaging, further supporting RH324’s anti-neoplastic effects.

Each SUV metric provides unique insights into tumor metabolic activity. SUV_max_ captures the highest uptake value, offering a measure of the most metabolically active portion of the tumor. SUV_mean_ represents the average uptake within the region of interest, providing a broader view of metabolic changes. Meanwhile, SUV_peak_ offers a balance between these 2 extremes, giving a more robust measure of metabolic response compared to SUV_max_ or SUV_mean_ alone. The complementary nature of these metrics strengthens the evaluation of treatment effectiveness.

Post-PVC adjustments further strengthened these findings by accounting for the PVE, which can obscure true metabolic activity in small or heterogeneous lesions. By correcting for these factors, the post-PVC values provide a more accurate representation of the tumor’s metabolic state, highlighting a clearer and more measurable metabolic response across all 3 metrics (SUV_max_, SUV_mean_, and SUV_peak_). This correction reduces the likelihood of underestimating the treatment’s effect, thus supporting the anti-neoplastic efficacy of RH324. This multi-metric approach, combined with post-PVC adjustments, underscores the importance of utilizing different SUV parameters to capture a comprehensive and precise assessment of treatment response.

The adoption of FDG PET/CT imaging in this study is predicated on the understanding that conventional anatomic imaging, governed by World Health Organization (WHO), RECIST, and RECIST 1.1 criteria, exhibits inherent limitations in accurately gauging the efficacy of contemporary cancer treatments, especially those that achieve disease stabilization rather than reduction in tumor size ^15^. In contrast, FDG-PET/CT provides a more detailed insight by providing the biological features of tumor lesions, such as changes in metabolism and intratumoral heterogeneity, which often precede measurable changes to the structure of tumor. To our knowledge, this study is the first ever in vivo human research to utilize FDG-PET/CT as an imaging biomarker of anti-neoplastic activity of RH324, a U.S. FDA-regulated drug product, used as a monotherapy in patients with refractory NSCLC.

In our study, we adapted PERCIST guidelines, which recommend using lesions larger than 15 mm in diameter on an axial slice of a registered CT image, as criteria for determining evaluable targets.^15^ The 15 mm in diameter equates to a volume of ~1.77 mL. To align with voxel spacing of the acquired PET images in this study, we selected 3 × 3 × 3 voxels, which equates to a volume of ~1.73 mL. Although the chosen volume threshold of our study was slightly smaller than the PERCIST criterion, this approach effectively captures the PVE and adheres to PERCIST, providing valuable insights into tumor heterogeneity and response to RH324 monotherapy in non-small cell lung cancer.

While the parameter values for the RL algorithm were carefully selected for PVC in this study, there remains scope for further optimization. The parameters chosen reflect a balance between accuracy and computational feasibility; however, parameter fine-tuning or the exploration of alternative algorithms could potentially enhance the efficacy of PVE reduction. Advances in algorithmic strategies and computational techniques may offer new pathways to improve PVC, thereby providing even more precise quantification of metabolic activity in PET imaging than the RL algorithm used in our study. Exploring these possibilities could yield significant improvements in the accuracy of PET-based assessments, particularly in the context of small lesion analysis where PVE is most pronounced.

There were several limitations of our study, including a small cohort size of 5 patients, which limited the generalizability of our results. Additionally, the absence of a placebo control group made it challenging to definitively attribute the observed effects to RH324. The lack of long-term follow-up also impeded our understanding of the treatment’s prolonged efficacy. Addressing these challenges in future studies will be crucial for a comprehensive evaluation of RH324’s anti-neoplastic potential in non-small cell lung cancer. Nevertheless, our results indicate the potential for RH324 and motivate a larger clinical trial.

## Conclusion

Our study demonstrates measurable response rates to oral RH324 monotherapy in advanced, refractory metastatic NSCLC, particularly in lesions with high metabolic activity as indicated by SUV_max_ values above 7.5 g/mL. In addition, there were no new metastatic lesions identified on post monotherapy FDG PET/CT imaging, further supporting RH324’s anti-neoplastic effects. However, the small sample size and absence of a control group in our study highlight the need for further research with a broader and more diverse cohort, as well as extended follow-up periods, to fully ascertain the therapeutic value and scope of RH324 in NSCLC management.

The utilization of FDG-PET/CT imaging, guided by adapted PERCIST criteria, provided a novel approach to evaluating the anti-neoplastic efficacy of RH324. This method offered deeper insights into tumor metabolism and heterogeneity, underscoring the potential of RH324 in treating refractory NSCLC.

Our study design sets the stage for assessment of many other traditional therapies using a short-term metabolic endpoint as a biomarker of potential clinical efficacy. As large randomized trials are generally not feasible with complementary and alternative therapies, strategies to assess clinical efficacy may benefit from non-invasive pharmacodynamic monitoring using a metabolic surrogate as a biomarker of disease activity.
